# Fabrication of triboelectric nanogenerators based on electrospun polyimide nanofibers membrane

**DOI:** 10.1038/s41598-020-59546-7

**Published:** 2020-02-17

**Authors:** Yeongjun Kim, Xinwei Wu, Je Hoon Oh

**Affiliations:** Department of Mechanical Engineering, Hanyang University, Ansan, Gyeonggi-do 15588 Republic of Korea

**Keywords:** Energy science and technology, Engineering, Materials science, Nanoscience and technology

## Abstract

Surface modification of polyimides (PIs) using electrospinning would significantly improve the performance of TENGs because of the larger surface area of the electrospun friction layer. However, PIs generally have high solvent resistance, so it is complicated to convert them into nanofibers using electrospinning process. This study aims to fabricate PI nanofibers via simple, one-step electrospinning and utilize them as a friction layer of TENGs for better performance. PI nanofibers were directly electrospun from PI ink made of polyimide powder without any additional process. The effect of PI concentration on spinnability was investigated. Uniform and continuous nanofibrous structures were successfully produced at concentrations of 15 wt% and 20 wt%. Electrospun PI nanofibers were then utilized as a friction layer for TENGs. A TENG with 20 wt% produced an open circuit voltage of 753 V and a short circuit current of 10.79 μA and showed a power density of 2.61 W m^−2^ at a 100 MΩ load resistance. During tapping experiment of 10,000 cycles, the TENG could stably harvest electrical energy. The harvested energy from the proposed TENG is sufficient to illuminate more than 55 LEDs and drive small electronic devices, and the TENGs exhibit excellent performance as a wearable energy harvester.

## Introduction

The rapid development of flexible electronics has promoted the wide application of various low power consumption electronic devices. Thus, an effective power source of these devices has gained increasing attention. Nanogenerators are good candidates due to their ability to harvest electrical energy sustainably from environmental sources^1–7^. Among various types of nanogenerators such as triboelectric, pyroelectric, thermoelectric, and piezoelectric nanogenerators, triboelectric nanogenerators (TENGs) have gained enormous attention over past few years owing to their simple configuration, low weight, and cost-effective fabrication process. TENG’s electrical performance is usually assessed by power density (W m^−2^), including voltage and current. TENG’s power density is relatively small compared to other types of nanogenerators because the current generated by TENGs is insufficient.

In order to enhance the performance of TENGs, various approaches have been conducted. Selecting materials of the friction layers according to the triboelectric series is an easy, reliable, and straightforward way. The farther two materials on the triboelectric series table, the higher the amount of charges generated. Many synthetic polymers have the nature of being negatively charged while nylon, cotton, and aluminum are generally used as positive friction layers. Polyimide (PI) has been widely used as a negative friction layer for TENGs due to its highly negatively charged nature^8–11^. In addition, PI exhibits excellent stability as a friction layer under repetitive external pressure or deformation due to its outstanding mechanical properties.

Some researchers have tried to further improve the performance of TENGs by enlarging the surface area of the friction layer with enhanced surface morphology^10,12–14^. They achieved relatively high performance, but either the complicated, expensive photolithography process or the E-beam evaporation method was used to fabricate the surface microstructures. Others developed porous structures inside the friction layer using pore foaming technology, such as particle templating methods^14–18^. This approach was cheap and easy to handle, but fabricated TENGs showed insufficient results. Thus, a simple and efficient way to enhance the surface area of the friction layer and hence improve the performance of TENGs has recently been required.

Electrospinning could be one of the best approaches for manipulating the friction layer because this technique is capable of producing long continuous nanofibers directly^19–23^. In electrospinning, a solution is ejected from a nozzle by applying a high electric potential difference between a nozzle and a collector. After ejection, the jet is elongated by whipping instability before deposition due to the interactions between the charges existing on the jet, resulting in continuous fiber structures. The electrospun nanofibers show a remarkably large surface area per unit volume and a rough surface as well. It has been found that electrospun nanofibers can improve the triboelectric effect, and they allow the generation of more charges^17,24–26^.

Therefore, electrospinning of PI nanofiber membranes would lead to the enhanced performance of TENGs because of both PI’s triboelectric nature and its enlarged surface area. However, PI has high solvent resistance, and they are typically insoluble^27,28^. In other words, it is challenging to fabricate electrospun PI nanofibers. To overcome this problem, PI nanofibers are commonly made through the following two-step approach: (1) electrospinning of polyamic acid (PAA) precursor via a polymerization process to form PAA nanofibers, (2) conversion of the PAA nanofibers into the PI nanofibers through thermal/chemical imidization process under several hundred degrees of Celsius for a few hours^24,29–31^. As expected, this two-step approach is complicated and expensive. Furthermore, since TENGs generally use flexible plastic substrates, high process temperature over a few hundred degrees of Celsius should be avoided. As a result, one-step, simple, direct electrospinning of PI nanofibers is crucial to fabricate more enhanced TENGs with higher performances.

In this study, we first developed a simple, one-step process for fabricating electrospun PI nanofibers from PI powder without any additional process. PI powders were dissolved into dimethylacetamide (DMAc), and the effect of PI concentration on the nanofiber structure was investigated. Then, TENGs were fabricated using electrospun PI nanofiber membrane. We investigated the influence of PI concentration on the performance of TENGs with contact-separation mode. The fabricated TENGs were also tested in various applications such as capacitor charging and LED illumination. It is expected that electrospun PI-nanofiber based TENGs could be utilized as an energy-harvesting device.

## Results and Discussion

### Electrospun PI nanofibers

As mentioned earlier, the two-step process is generally used to fabricate electrospun PI nanofibers due to PI’s high solvent resistance. PAA monomers are first electrospun to fabricate the nanofibrous structure, and they are converted into PI through imidization process. In contrast, PI powder is soluble in DMAc, which is one of an organic polar solvent, so it is worthwhile to investigate whether direct electrospinning of PI solution is applicable. Since direct electrospinning does not require thermal/chemical post-processing, it has an advantage in the fabrication of TENGs with a flexible plastic substrate. The PI friction layer with electrospun nanofiber structures has a much larger surface area than film structures, so TENGs with nanofiber membrane would produce more enhanced electrical performance.

**Figure 1:**
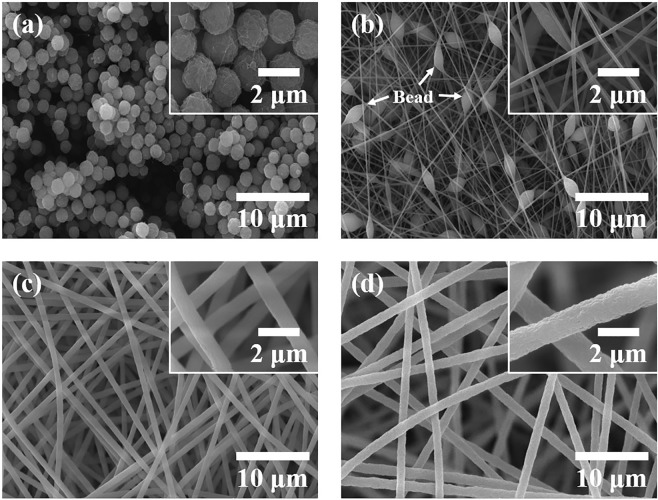
Effect of PI concentration on the microstructures of electrospun nanofiber membranes: (**a**) 5 wt%, (**b**) 10 wt%, (**c**) 15 wt%, and (**d**) 20 wt%.

In order to fabricate nanofibrous membranes using electrospinning technique, we first investigated the solubility and spinnability of the PI powders with various solvents (See Supplementary Information Figs. S1 and S2). Among various solutions, PI powder in DMAc showed good solubility, spinnability, and performance as a material for the friction layer of TENG (Supplementary Information Fig. S3). We then examined the effects of the PI concentrations in inks on nanofiber morphology. Figure 1 depicts the SEM images of the electrospun PI membranes. For 10 wt%, 15 wt%, and 20 wt% PI solutions, typical continuous nanofibrous structures were obtained. The diameter of the nanofibers tended to increase with increasing PI concentration. Uniform nanofibrous structures were formed with 15 wt% and 20 wt%, but a beads-on-string structure was exhibited with the 10 wt%. Moreover, there was no fibrous structure with the 5 wt%, which means that it was electro-sprayed. This might be a result of low cohesion force between the polymer chains in the solution^32,33^. The solution properties such as polymer concentration and molecular weight of the ejected polymer significantly affect fiber formation in comparison to other governing parameters like surface tension and conductivity. For low molecular weight polymers, a high polymer concentration is necessary to fabricate uniform nanofiber structures. In other words, once the polymer and the solvent capable of electrospinning are determined, the characteristics of the electrospun nanofibers are mainly influenced by the polymer concentration in ink.

In this work, uniform and continuous electrospun PI nanofibers could be produced with concentrations from 15 wt% and 20 wt%. However, over PI concentration of 20 wt%, PI powders were not completely dissolved in DMAc, resulting in agglomerated solutions.

### Evaluation of TENGs

**Figure 2:**
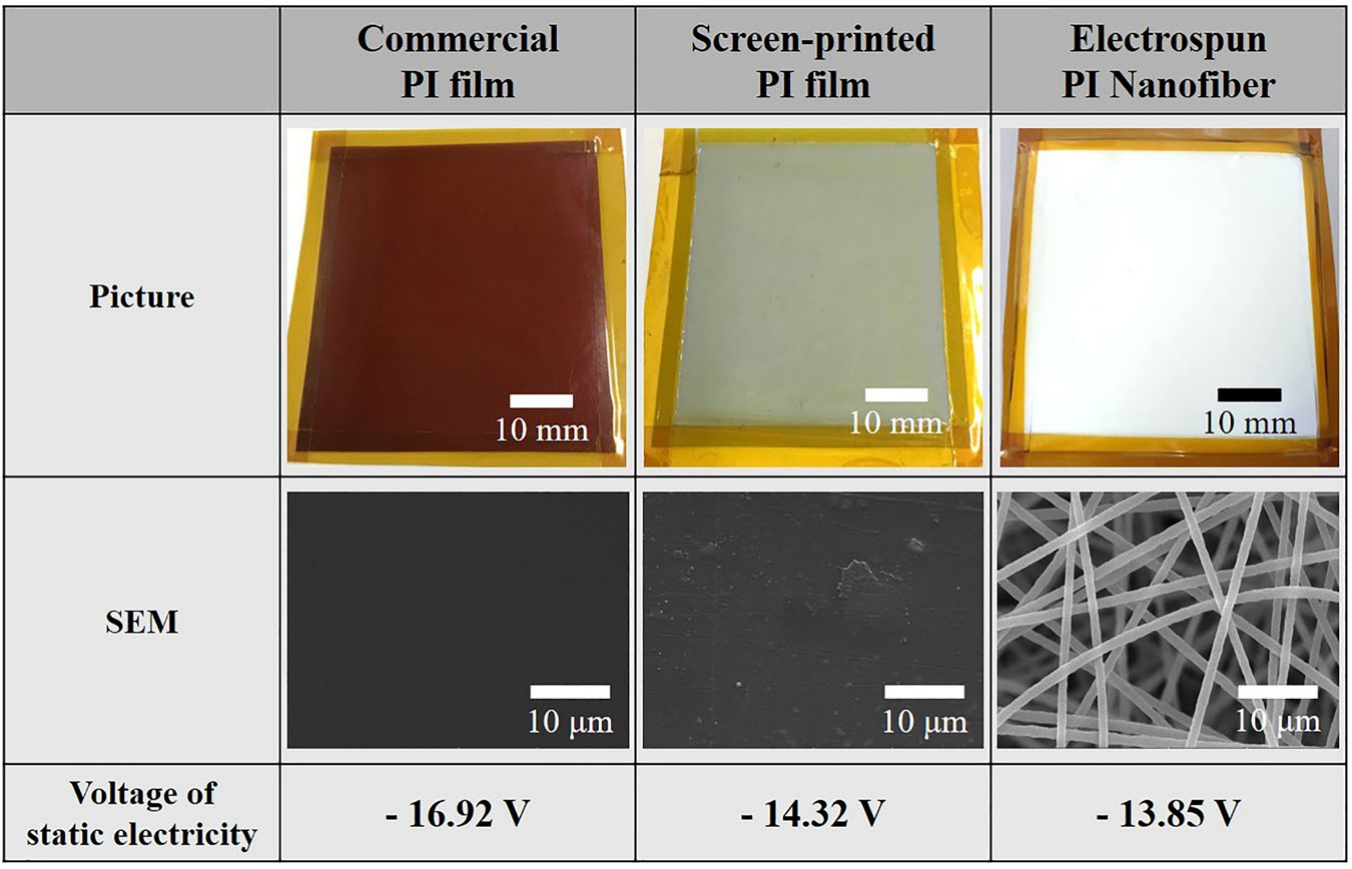
Types of friction layers: commercial PI film, screen-printed PI film and electrospun PI nanofiber membrane. Photographs, SEM images and the voltages of static electricity on the surface of three PI layers.

We fabricated TENGs by using the electrospun PI nanofibers of a 15 wt% concentration as a friction layer and prepared two more friction layers such as a commercial PI film and a screen-printed PI film for comparison purpose (Fig. 2). As shown in the SEM images in Fig. 2, the surface morphology of commercial PI film and screen-printed PI film were almost flat. The surface area of these flat surfaces would be much smaller than that of electropsun PI nanofibers. To investigate the voltages of static electricity of the film surface in the neutral state, PI films were left at the room temperature with the humidity of 28% for 24 h to remove any effect on static electricity from the fabrication process like electrospinning. The voltage of static electricity on the surface of the samples were then measured using an electrostatic field meter (FMX-004, SIMCO), and the values of each sample were almost the same in all three films (Fig. 2). In particular, the measured values of the screen-printed PI film and the electrospun PI nanofibers fabricated using the same material showed only a little difference. Since the materials of the two films were the same, and the voltages of static electricity were obtained from the outermost surface of the film, the two values were almost similar. However, electrospun PI nanofibrous membrane would perform much better as a friction layer of a TENG because there is a surface to which charges can be induced to the fibers inside the membrane as well as the surface. When a commercial PI film was used as a friction layer, its four sides were firmly adhered to the ITO-PET bottom electrode using Kapton tape. In order to evaluate voltage, current, and power density of each sample, contact and separation cycles were repeated 20 times for each sample, and these values were averaged. The compressive force of ~10 N was applied at a frequency of 2 Hz.

**Figure 3:**
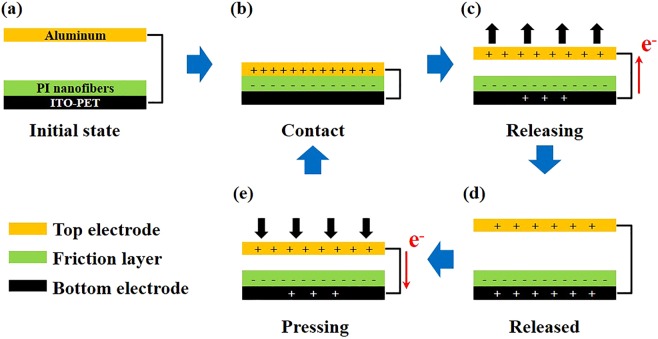
Illustration of the operating mechanism of contact-separation mode TENGs: (**a**) initial, (**b**) contact, (**c**) releasing, (**d**) fully released, and (**e**) pressing states.

All TENGs were evaluated and characterized in contact-separation mode^10^. Figure 3 shows a schematic illustration of the operating principle of a typical TENG. The TENG mechanism is based on the coupling effect of triboelectrification and electrostatic induction. At the initial state, there is no electric charge in the layers because there is no contact between the nanofibers and the top electrode (Fig. 3a). When the two layers come into contact, they exchange free electrons based on their electron affinities. In this state, all charges are in the same plane; thus, the TENG is in an electrically neutral state (Fig. 3b). When the top electrode is separated, the device is no longer in electrical equilibrium, allowing the free electrons in the bottom electrode attached to the top electrode to move toward equilibrium (Fig. 3c,d). When the device is pressed again, the free electrons return to their original position (Fig. 3e). Based on this process, AC electrical output is exhibited, and voltage and current are observed depending on the electrical wiring of a load resistor between the two electrodes.

For evaluating the performance of TENGs, power generation is defined by three parameters: the voltage (*V*) between the electrodes, the amount of transferred charge (*Q*) in between, and the separation distance (*x*) between the two triboelectric charged layers (in this case, the electrospun PI nanofibers and aluminum electrode). The output voltage of a typical TENG operated in contact-separation mode is determined as follows^6^:

1 $$V=\frac{{\rho }_{A}\cdot x(t)}{{\varepsilon }_{0}}-\frac{Q}{S{\varepsilon }_{0}}(\frac{d}{{\varepsilon }_{r}}+x(t))$$

where *ρ*_*A*_, *ε*_*r*_, and *S* are the charge density on the surface of the contact materials, the relative permittivity of friction layer, and the surface area, respectively. In addition, *d* and *x(t)* denote the thickness of the friction layer and the distance variation between the contact materials, respectively. In the open circuit condition, the output voltage is linearly proportional to the product of *ρ*_*A*_ and *x(t)* because *Q* is zero. When we define the effective thickness constant *d*_0_ as *d/ε*_*r*_, the output current in the short circuit is highly proportional to the *S* and *ρ*_*A*,_ as follows:

2 $${I}_{SC}=\frac{S{\rho }_{A}{d}_{0}v(t)}{{({d}_{0}+x(t))}^{2}}$$

**Figure 4:**
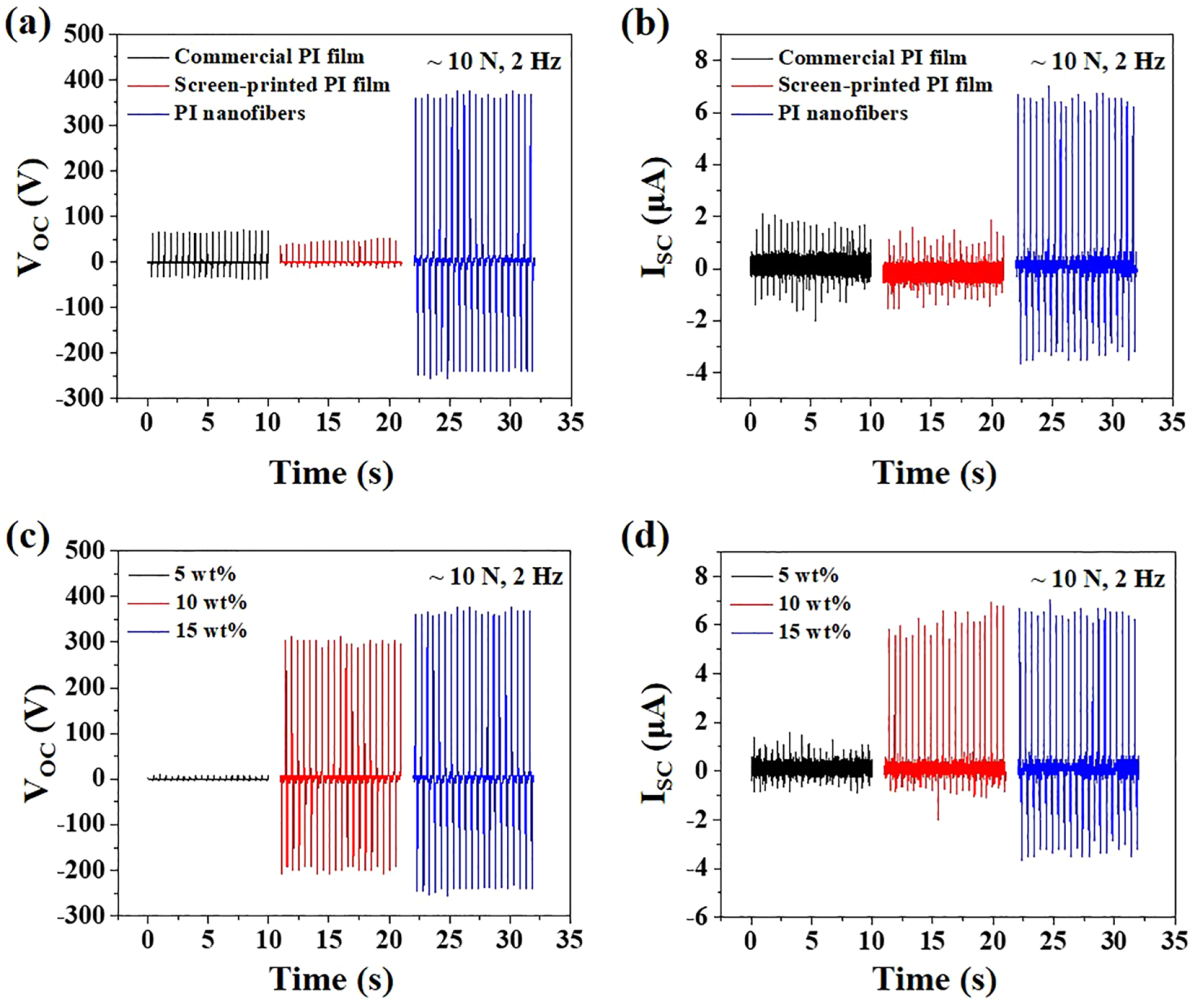
Effect of friction layer types on (**a**) V_OC_ and (**b**) I_SC_. Effect of PI concentrations on (**c**) V_OC_ and (**d**) I_SC_.

We measured both the V_OC_ and the I_SC_ of the TENGs. As we expected, the TENG with PI nanofibers showed much higher electric performance, as shown in Fig. 4a,b due to its high surface area. Charges in the nanofibrous membrane are induced not only on the surface of the membrane but also on the surfaces of the inner fibers by electrostatic induction. Therefore, *ρ*_*A*_ is greatly enhanced with nanofibrous structures compared to film like structures. The V_OC_ values for the TENGs with the commercial PI film, the screen-printed PI film, and the electrospun PI nanofibers were 66.1 V, 45.6 V, and 366 V, respectively. The I_SC_ showed the same tendency, and the measured values were 1.68 μA, 1.61 μA, and 6.52 μA for the commercial PI film, screen-printed PI film, and electrospun PI nanofibers, respectively. It should be noted that the voltages of static electricity on the surfaces were almost the same among all three PI films.

**Figure 5:**
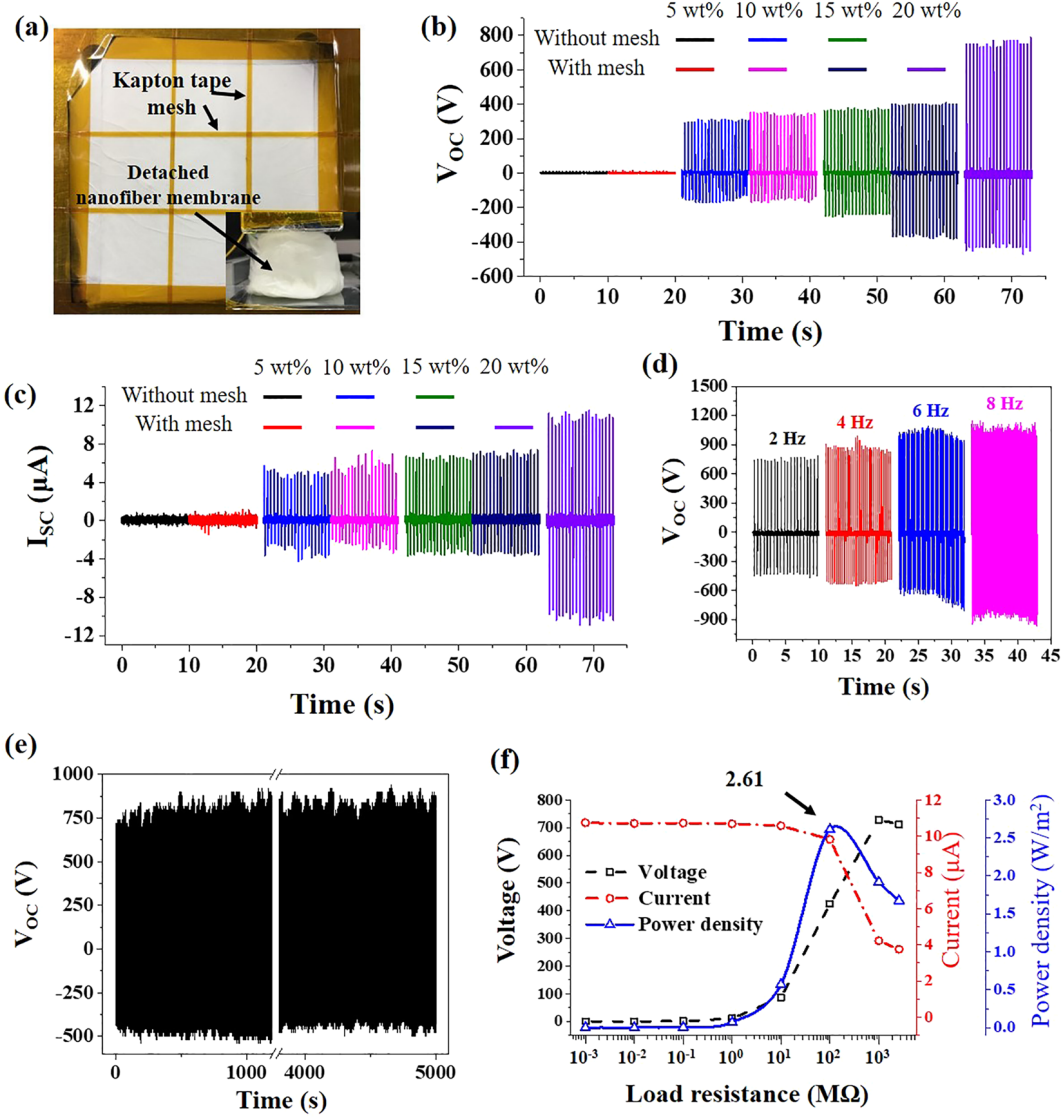
(**a**) An image of PI nanofiber membrane with reinforcing Kapton tape mesh. The inset image shows a detached PI nanofiber membrane without the Kapton mesh during measurement. (**b**,**c**) Effect of mesh on VOC and ISC. (**d**) Effect of tapping frequency on VOC with meshed 20 wt% PI nanofiber membrane. (**e**) Continuous VOC output with a motorized tapping machine for 10,000 cycles under pressing force of ~10 N. (**f**) Voltage, current, and power density of the TENG with Kapton-meshed 20 wt% PI nanofiber membrane.

We then investigated the effects of the PI concentration on the output performance of TENGs. The thickness of the friction layers were measured using a micrometer. The measured thickness of the PI friction layers using 5 wt%, 10 wt%, 15 wt%, and 20 wt% ink were 17 μm, 33 μm, 52 μm, and 64 μm, respectively. As shown in Fig. 4c,d, both V_OC_ and I_SC_ improved as the PI concentration increased. The variation of *x(t)* due to different *d* is too small to have an influence on output voltage. Therefore, the thickness of the friction layers has little impact on the electrical performance of TENGs. The low performance at 5 wt% was due to the structures of the layer. When the 5 wt% ink was used, it was sprayed instead of spun, and the PI was in the form of granules rather than nanofibers. The film was severely damaged during the measurement since the adhesion among the granules was very weak. The damaged PI film was represented in Supplementary Information Fig. S4. On the other hand, the layers that were electrospun with 10 wt% and 15 wt% possessed continuous nanofiber structures, resulting in much higher electrical performances. The V_OC_ values for the TENGs with 10 wt% and 15 wt% were 300.8 V and 366 V, respectively. The I_SC_ values were 6.15 μA and 6.52 μA, respectively. However, we were not able to measure the corresponding values of the TENG with 20 wt% because the layer was detached entirely from the ITO-PET at the very beginning of the measurement (Fig. 5a). From the tendency of the TENGs with 5 wt% through 15 wt%, it can be expected that the TENG with 20 wt% would show the highest performance if it is firmly attached to ITO-PET.

In order to prevent the friction layer with 20 wt% from falling off the ITO-PET, we reinforced the layer by applying Kapton tapes on it in mesh form (Fig. 5a). Kapton is one of the PIs, and it is assumed that the Kapton mesh fixture would not significantly affect the electrical performance of the TENGs. The width of the mesh was 1 mm, and the distance from each mesh was 13 mm. The electrical performance of TENG reinforced with Kapton mesh was measured without any difficulties. Moreover, TENGs fabricated with 5 wt%, 10 wt%, and 15 wt% inks were also reinforced with Kapton mesh and compared with those without Kapton mesh to investigate the effect of the mesh on the electrical performance of TENGs. Both V_OC_ and I_SC_ remarkably increased as PI content increased from 5 wt% to 10 wt% and 15 wt% to 20 wt% (Fig. 5b,c). As mentioned above, the PI film fabricated with 5 wt% ink was seriously damaged during measurement and show very low electrical performance. The V_OC_ of the TENGs with 15 wt% and 20 wt% inks increased about 190% from 398 V to 753 V, and the I_SC_ increased from 6.95 μA to 10.79 μA. With a higher polymer concentration in the electrospun inks, both fiber diameter and surface area tend to increase^34,35^. Thus, more charges would be generated with higher surface area, resulting in higher electrical performance. In addition, there was a negligible difference in performance due to the presence of the mesh as expected. This might be because the Kapton mesh and PI nanofibers are almost in the same position on the triboelectric series, and the difference in the surface area between meshed- and non-meshed friction layers was not sufficient to affect the performance of the TENGs.

We evaluated the performance of the TENG with 20 wt% concentration and mesh. Figure 5d shows the effect of tapping frequency on the V_OC_. The V_OC_ of the TENGs was remarkably enhanced from 753 V to 1,073 V as the frequency was increased. At faster tapping frequency, the external electrons flow to reach an equilibrium in less time^13,36,37^. In addition, as the frequency increases, the pushing force increases, thereby enhancing the contact area and improving the V_OC_ (See Supplementary Information Fig. S5)^11,14^. The TENG with mesh also showed excellent robustness (Fig. 5e). It was continuously pressed and released for 5,000 seconds at 2 Hz, and it showed almost the same V_OC_, approximately 750 V, during the entire cycles. The electrospun PI nanofibers were almost not damaged during the cyclic test due to its excellent material property (Supplementary Information Fig. S6). For practical applications, we measured the power density under load resistance. The TENG was connected to resistors, with resistance values ranging from 1 kΩ to 2.6 GΩ. As shown in Fig. 5f, the output voltage increased with increasing load resistance, and the output current showed the opposite tendency. A maximum power density of 2.61 W m^−2^ was achieved at a load resistance of 100 MΩ.

### Demonstration of TENGs

**Figure 6:**
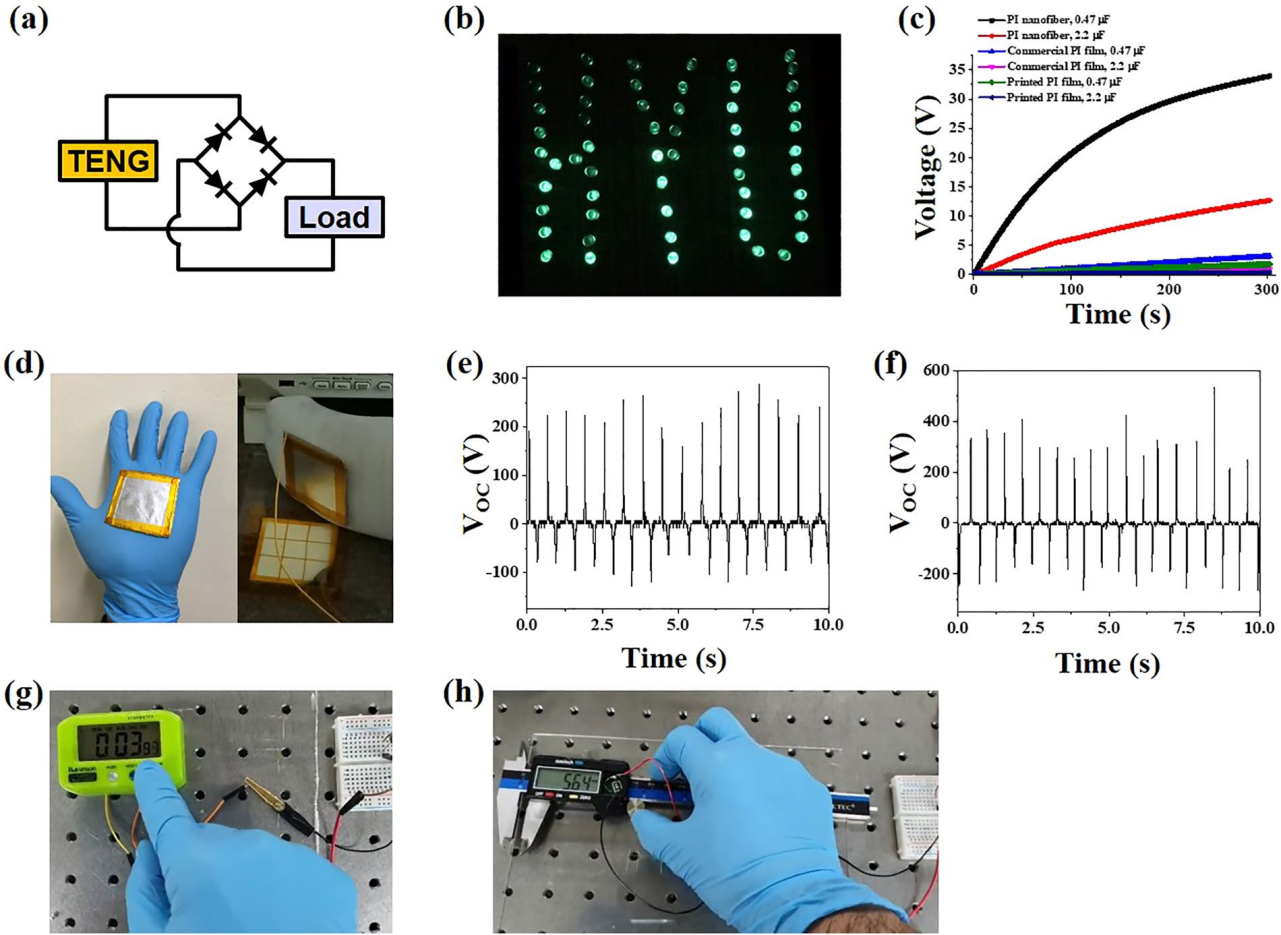
Demonstration of the best TENG in this study: (**a**) electric circuit for various applications, (**b**) illumination of 55 LEDs, and (**c**) charging of various capacitors with different types of PI dielectric layer. (**d**) Photographic image of the electrode and TENG devices to harvest electric energy from human motions, and the voltage output of (**e**) palm and (**f**) foot tapping. Use of the TENG to turn on the devices such as (**g**) a stopwatch and (**h**) a digital Vernier caliper.

Several confirmatory experiments were carried out for future applications. Figure 6a shows a full-wave rectifier bridge to convert AC output to DC output. The TENG was connected with 55 commercial green light-emitting-diodes (LEDs) which have a turn-on voltage of ~3 V. All the LEDs assembling the word “HYU” could easily be lit up by the TENG under the frequency of 2 Hz (Fig. 6b and Supplementary Information Video S1). At the same operating conditions, a 0.47 μF and 2.2 μF capacitors were charged to 33.8 V and 12.6 V, respectively, within 300 seconds (Fig. 6c). On the other hand, when a commercial PI film and a screen-printed PI film were used, 0.47 μF capacitors were charged to 3.09 V and 1.73 V, respectively. For 2.2 μF capacitors, the charged voltages were observed as 0.956 V and 0.255 V. It means that the electrospun PI nanofibrous membrane is more advantageous for charging capacitors due to its much-enlarged surface area. We also demonstrated the dynamic energy harvest with the palm- and the foot-attached devices. As shown in Fig. 6d, we placed 40 mm by 40 mm aluminum electrodes on the palm and the foot and consecutively tapped an electrospun PI nanofiber membrane. As can be seen from Fig. 6e,f, the TENG harvested electric energy from each tap in a stable manner. The voltages produced by TENGs attached to the palm and the sole were ~250 V and ~300 V, respectively (See Supplementary Information Videos S2 and S3). The TENG attached to the palm produced relatively low voltage because pressing force was weak than that of the foot. However, it was confirmed that both TENGs could stably harvest electric energy from TENGs which were attached to the curved human body. In addition to energy harvest from the human motions, the proposed TENG could turn on small electronic devices such as a stopwatch and a digital vernier caliper by charging a commercial capacitor of 330 μF to 16 V. As shown in Fig. 6g,h, the TENG could sufficiently drive the devices (see Supplementary Information Videos S4 and S5). The TENG has good energy harvesting capability and hence great potential in the field of wearable devices.

## Conclusion

We directly fabricated PI nanofibers using PI powder via an electrospinning technique without any post-process such as imidization. Uniform and continuous nanofibrous structures were successfully produced with PI concentrations of 15 wt% and 20 wt% in DMAc. When the electrospun PI nanofiber was used, we found that the voltage was increased by a factor of approximately 5.5, and the current was improved by a factor 3.9 in comparison with the film-like structures. However, when the 20 wt% ink was used, there was a problem, in which the PI nanofiber layer was detached from the ITO-PET substrate during the measurement. In order to solve this problem, Kapton tape was used to reinforce the PI nanofiber layer in the form of a mesh, and it was confirmed that a voltage of approximately 753 V was generated. The TENG with 20 wt% was able to stably harvest electric energy during the tapping experiment of 10,000 cycles and even from finger and foot tapping. The proposed PI nanofiber-based TENG could be utilized in various applications, such as capacitor charging, LED illumination and driving of small electronic devices.

## Methods

### Materials

To prepare electrospinning PI inks, commercial polyimide powder (PI resin powder, Alfa Aesar) was dissolved in dimethylacetamide (DMAc, Sigma Aldrich) for 12 h using a magnetic stirrer at room temperature. The concentration of PI varied from 5 to 20 wt% with an interval of 5 wt%. An indium-tin-oxide coated polyethylene terephthalate (ITO-PET) was used as a collector. Aluminum was used as a top electrode of the TENGs.

### Electrospinning process

Electrospinning was performed using an in-house system (Supplementary Information Fig. S7). A 27 G metal nozzle (with an inner diameter of 210 μm) was connected to an adaptor acting as an upper electrode, and a high-voltage supplier (FJ50P2.5, Glassman) was used to apply high electric potentials to the nozzle and PI ink. ITO-PET substrate (40 × 40 mm^2^) was ultrasonicated for 5 min in deionized water to remove contamination on the surface. ITO-PET was placed on the moving stage, and electrospun PI nanofibers were collected on the ITO-PET substrate. The distance from the nozzle tip to the surface of the collector was fixed at 150 mm. PI ink was fed into the nozzle using a syringe pump (PHD Ultra, Harvard Apparatus) with a fixed flow rate of 6 μl min^−1^. The input voltage was set at 10 kV, and PI electrospinning was performed for 1 h for all the experiments in this study. The surrounding humidity was kept below 35% by using a dehumidifier (NE-45ND, Nawoo).

### Fabrication and evaluation of PI nanofiber-based TENG

The electrospun PI nanofiber membranes on the ITO-PET were used as a friction layer for TENGs. The contact and separation process was conducted by the in-house actuating system controlled via LabVIEW (Supplementary Information Fig. S8a). ITO-PET, which was acted as a collector for the electrospinning process, was used as a bottom electrode for TENGs, and the top electrode was an aluminum tape (Supplementary Information Fig. S8b).

To compare film-like structures and nanofibrous structures, a commercial PI film and a screen-printed PI film were also prepared as friction layers for the TENGs. To fabricate the screen-printed PI film, PI ink with a concentration of 15 wt% was directly screen-printed onto the ITO-PET film using a razor blade and was heated to 60 °C for 3 h. The pressing force was measured with a load cell (UMM-K20, Dacell) and a data acquisition board (PXIe-4330, National Instruments). The electrical performance parameters, including the open circuit voltage (V_OC_) and the short circuit current (I_SC_), were measured using an oscilloscope (MDO-3012, Tektronix) and a preamplifier (SR570, Stanford Research Systems). The microstructure of the electrospun PI nanofibers was observed using a field-emission scanning electron microscope (FE-SEM: S-4800, Hitachi).

## Supplementary information

Supplementary Information.

Supplementary Information 2.

Supplementary Information 3.

Supplementary Information 4.

Supplementary Information 5.

Supplementary Information 6.
