# Supplementary material for: Fabrication of triboelectric nanogenerators based on electrospun polyimide nanofibers membrane
Source: Sci Rep. 2020 Feb 17;10:2742. doi: 10.1038/s41598-020-59546-7 (PMC7026082; doi:10.1038/s41598-020-59546-7)
Supplement: Supplementary file 1 — Supplementary Information. [file 41598_2020_59546_MOESM1_ESM.pdf]

Supplementary Information

# Fabrication of triboelectric nanogenerators based on electrospun polyimide nanofibers membrane

Yeongjun Kim[1], Xinwei Wu[1], and Je Hoon Oh[1,*]

[1] Department of Mechanical Engineering, Hanyang University, Ansan, Gyeonggi-do 15588, Republic of Korea

*Corresponding Author
E-mail: jehoon@hanyang.ac.kr

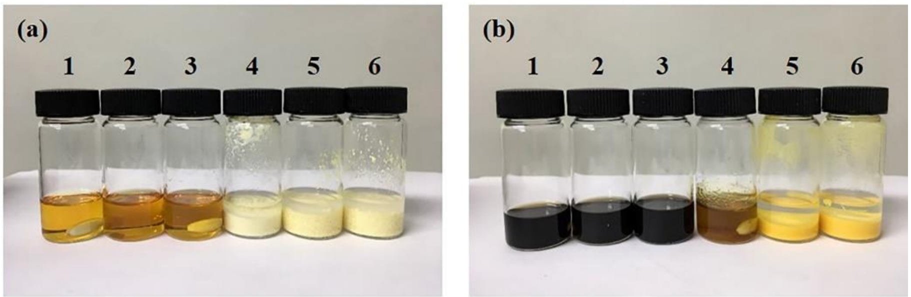


* 1: Dimethylacetamide (DMAc), 2: Dimethylformamide, 3: Dimethyl sulfoxide (DMSO), 4: Acetone, 5: Isopropyl alcohol, 6: Ethyl alcohol

**Figure S1.** Solubility of (a) the PI resin powder (Alfa Aesar) and (b) the PI plastic powder (VETC$^{TM}$) in various solvents.

We checked the solubility of a PI powder purchased from Alfa Aesar (PI resin powder, hereinafter referred to as PIr) and a powder purchased from VTEC$^{TM}$ (PI plastic powder, hereinafter referred to as PIp) in dimethylacetamide (DMAc), dimethylformamide (DMF), dimethyl sulfoxide (DMSO), acetone, isopropyl alcohol, and ethyl alcohol. All solutions were fabricated with 15 wt%, and electrospinning was conducted using the same fabrication parameters in the manuscript. Both powders showed relatively good solubility in DMAc, DMF, and DMSO (Figure S1). On the other hand, the powders were not fully dissolved and settled in powder form when acetone, IPA, and ethyl alcohol were used as solvents.

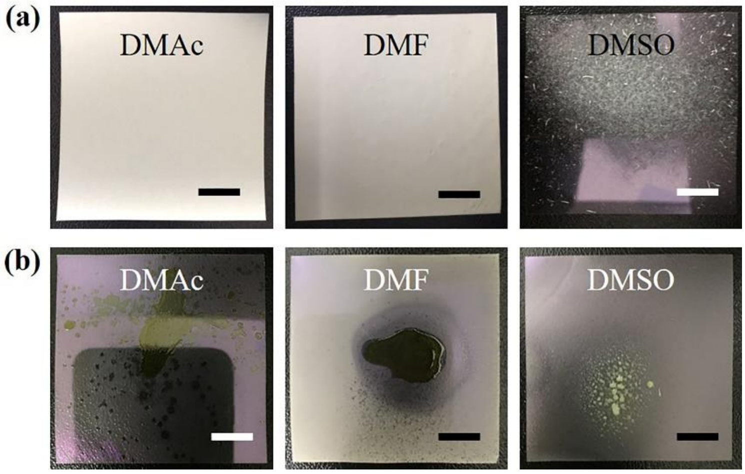


**Figure S2.** Spinnability check with (a) the PIr inks and (b) the PIp inks. Scale bars are 10 mm.

We then conducted electrospinning experiments of PI inks based on DMAc, DMF, and DMSO solvents. Figure S2(a) shows the electrospun layer using the PIr inks. The typical nanofiber membranes could be obtained when DMAc and DMF were used as solvents. However, PIr ink from DMSO showed poor spinnability. When DMSO was used, rod-shaped fibers were randomly sprayed on the substrate. This is probably due to insufficient viscosity and molecular weight of the solution. When the PIp inks were used, all of the solvents resulted in poor spinnability (Figure S2(b)). It might be caused by the same reason as mentioned above. As a result, the fabrication of nanofiber membranes through electrospinning was possible only when the PIr was dissolved in DMAc or DMF.

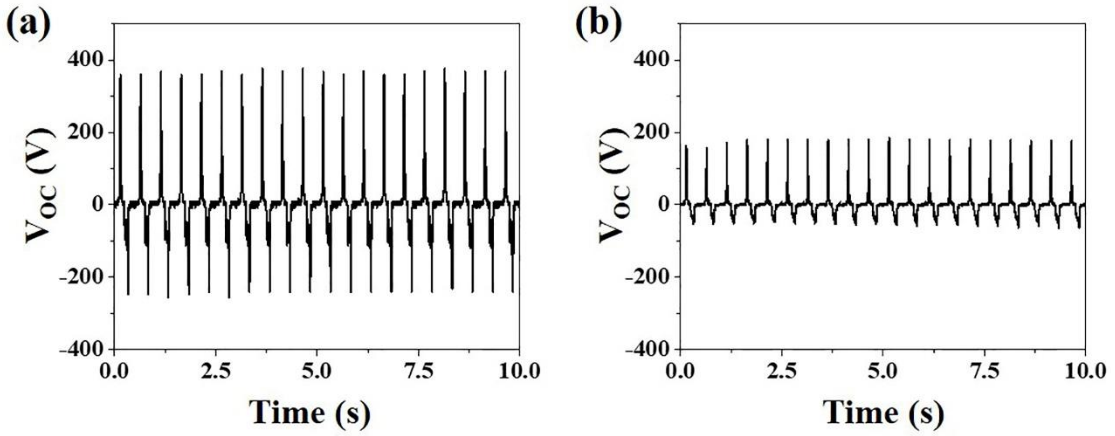


**Figure S3.** Open circuit voltage of TENGs when the PIr dissolved in (a) DMAc and (b) DMF.

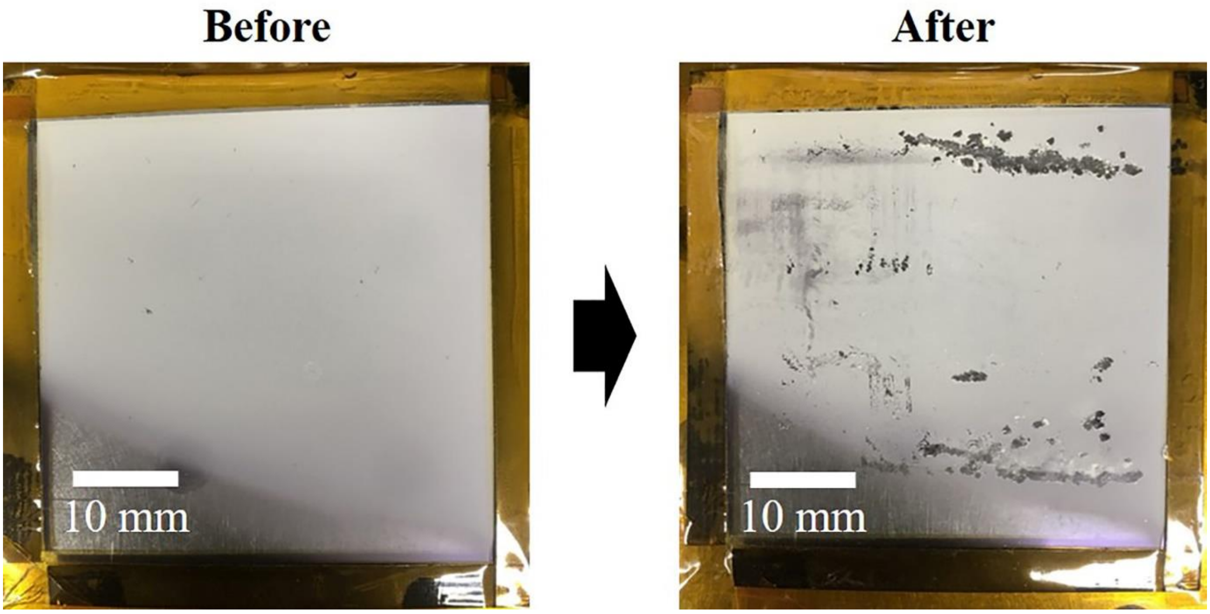


**Figure S4.** Images of electrosprayed PI film fabricated with 5 wt% ink before and after contact-separation experiment with 2 Hz and ~ 10 N for 1 s.

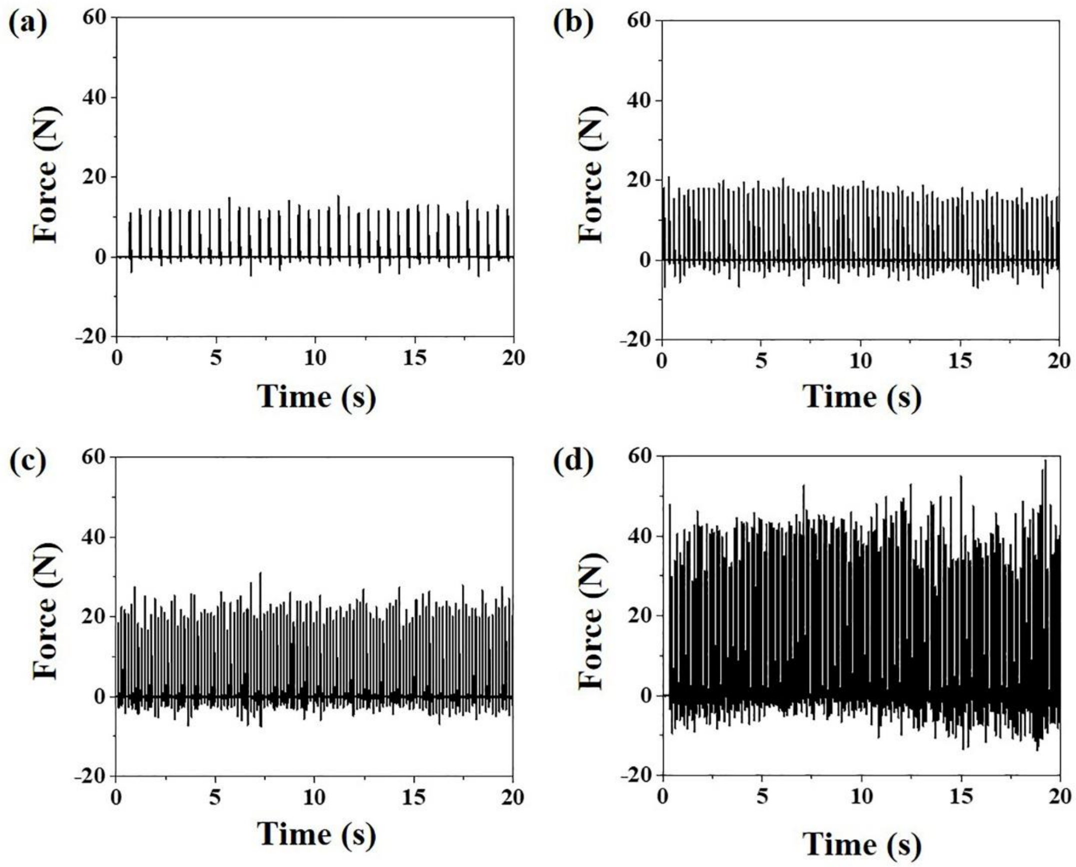


**Figure S5.** Force exerted on the TENG when the tapping frequency was (a) 2 Hz, (b) 4 Hz, (c) 6 Hz, and (d) 8 Hz.

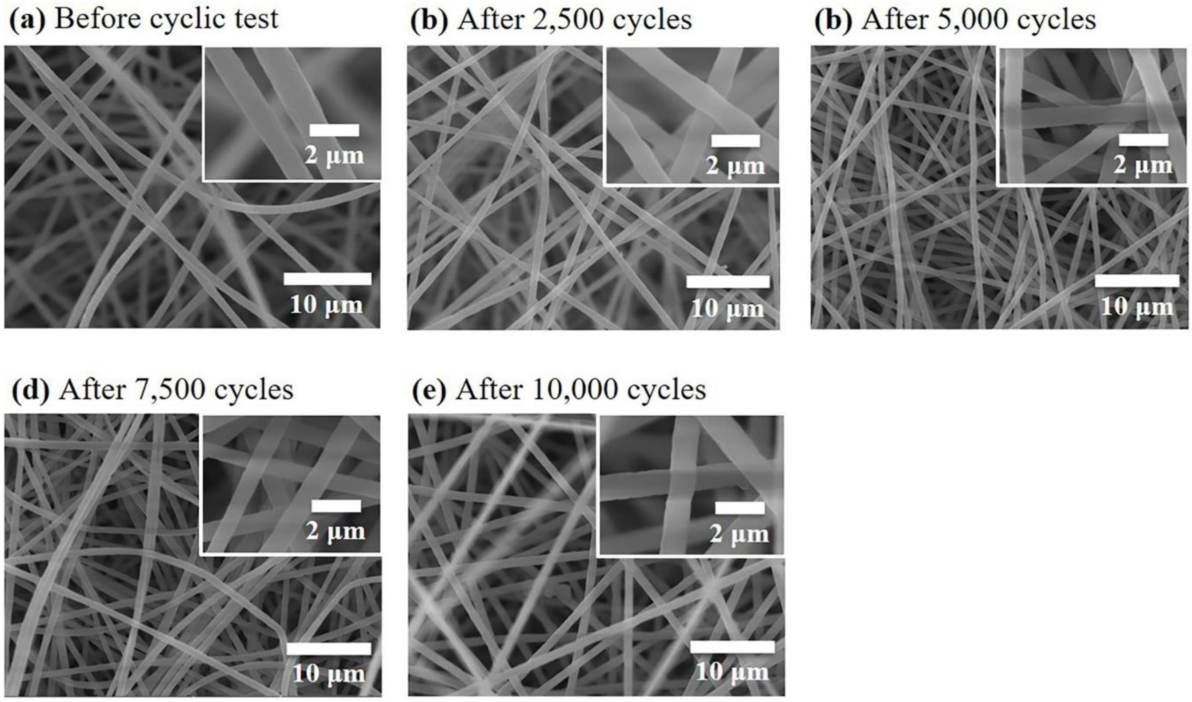


**Figure S6.** (a) The surface morphology of the electrospun nanofibers before cyclic test; and after (b) 2,500 cycles, (c) 5,000 cycles, (d) 7,500 cycles, and (e) 10,000 cycles.

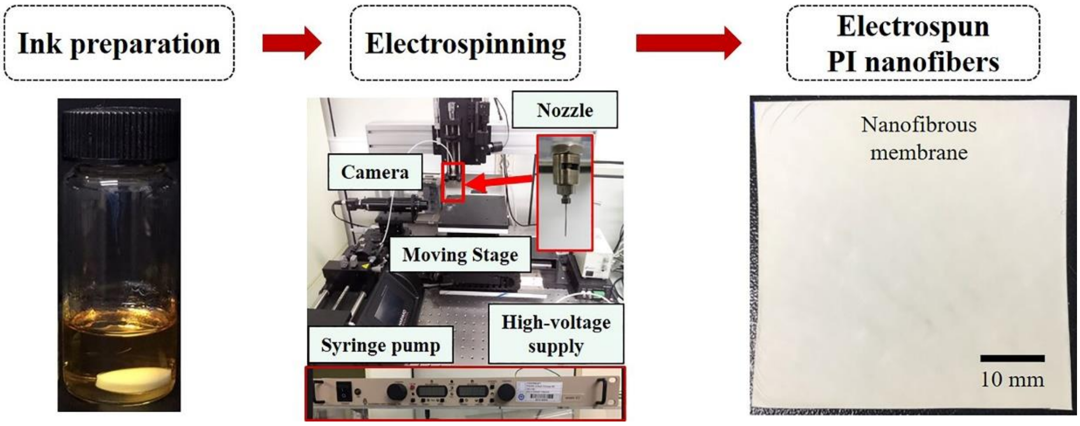


**Figure S7.** Illustration of fabrication procedure for PI nanofiber based TENG.

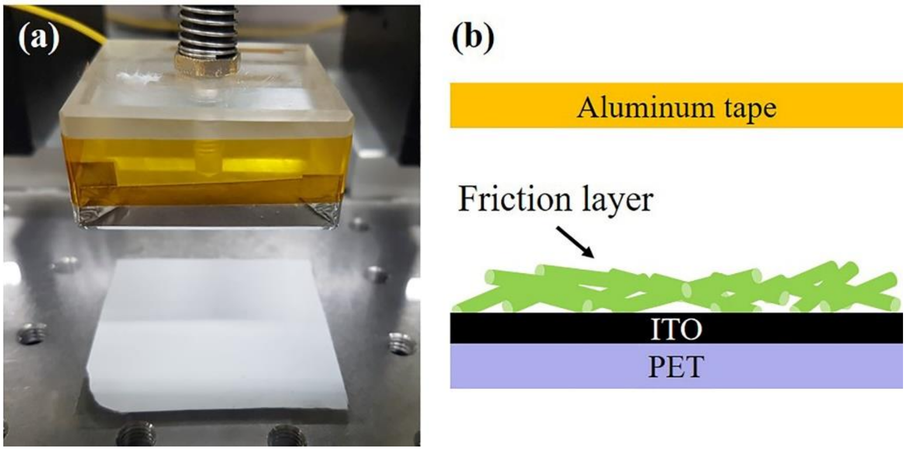


**Figure S8.** (a) In-house actuating system and (b) a schematic illustration of TENG.
